# Round the Hospitals

**Published:** 1920-04-24

**Authors:** 


### ROUND THE HOSPITALS.

Queen Alexandra received Sir Harold Boulton, Chairman of the Council of Queen Victoria's Jubilee Institute for Nurses, and Miss Peterkin, the General Superintendent of the Queen's Nurses, at Marlborough House, on April 16, when Her Majesty personally presented to Miss Peterkin the gold badge of the Institute in recognition of her long and distinguished services in connection with it. Miss Peterkin was enrolled as a Queen's Nurse on January 1, 1894, and held successively the posts of superintendent, inspector, and Superintendent for Ireland and Scotland before being promoted to her present office in 1917. The work of the Institute is increasing rapidly, and the nurses working under it now number 4,500. The energy, wisdom, and administrative ability of Miss Peterkin are

**ROUND THE HOSPITALS—*(continued)*.**

gratefully appreciated by her fellow-workers, and Queen Alexandra's recognition of the admirable work she is doing will give pleasure to all Queen's nurses.

The Jubilee Institute lost a sound friend in the passing of Adeline, Duchess of Bedford, whose requiem was sung on April 17, at St. Alban's, Holborn. The late Duchess was for some years Chairman of Queen Alexandra's Committee of 200, each member of which undertakes to give or provide £10 towards the Q.V.J.I.N. It devolves on the Chairman to keep the numbers up to strength, so the appointment is no sinecure. The Duchess will chiefly be remembered in connection with the subject of prison reform. She was one of the first lady visitors to convict prisons, and as Chairman of the Board of Visitors of Aylesbury Prison was instrumental in procuring the appointment of a medical woman as deputy-governor. The extension of the Borstal detention system to women was largely due to her influence.

A very interesting double ceremony, the first of its kind, took place at the Royal Infirmary, Liverpool, recently. The annual show of linen and other articles contributed by the Ladies' Linen League, now in its second year, was combined with a public presentation of prizes to the nurses who stood highest in the examinations. The Lady Mayoress, Mrs. Burton Eills, opened the exhibition of needlework contributed by the League, which totalled close on 800 articles, blankets, bed-linen, dressing-gowns, jackets, etc. Mr. Wade Deacon, speaking on behalf of the infirmary, forecasted the launching of an appeal in the near future for a new Nurses' Home, estimated at present prices to cost not less than £100,000. The Lady Mayoress distributed prizes to the successful candidates as follows:—*Special Subjects*, first, Nurse Davies; second, Nurse Robb. *Medical*, first, Nurse Newton; second, Nurse Bowden. *Surgical*, first, Nurse Nugent; second, Nurse Cooper. *Anatomy and Physiology*, first, Nurse Garnett; second, Nurse Bull. The Matron's Prize went to Nurse Nolan. The general high level of excellence reached by the nurses in all the examinations was matter of general remark, and reflected great credit on the two home sisters who did the coaching. At the conclusion of the proceedings Miss Cummins held a reception, when tea was served.

Miss Beatrice Cutler, for fifteen and a half years superintendent of the Nurses' Home, and assistant-matron at St. Bartholomew's Hospital, has been granted a pension of £84 a year on her retirement. This sum is approximately 20½/84ths of her average salary and emoluments for the past three years, the Committee having added in computing the pension five years to Miss Cutler's period of service as permitted by Clause 4 of the Pension Scheme "in the case of exceptionally efficient service." Miss Cutler will be very much missed by her colleagues, among whom she is highly popular. Her successor, Miss Helen I. Baines, whose appointment we announced last week, at present holds the position of Matron's Office Sister. The salary attaching to the post of assistant-matron at St. Bartholomew's is £110 per annum increasing £10 every two years to a maximum of £150, with board, residence, uniform and washing.

A further advance was made on the 15th inst. in the vital matter of forming an International Health Council under the League of Nations. At a luncheon given at the Carlton Hotel to representatives of the Allied nations Dr. Addison presided, and in proposing the toast of "The International Health Council" he expressed the hope that that body would in time be capable of rendering infinite service to mankind. Its opportunities for rendering useful service are limitless. They include not only the prevention of disease in communities, but the bettering of industrial conditions. There can be no doubt in the minds of close observers that men and women pursue their callings in crowded cities in every country of the world under conditions which tend to break down their normal resistance to disease. Systematic research into the causes which first induce ill-health can do much to point out the best line of action in prevention. When that has been ascertained the impetus afforded by combined international action is incalculable.

A beautiful tribute to Edith Cavell's memory has been laid at the base of her statue at Charing Cross by the Association Dames de France, in connection with the Croix Rouge Français. It takes the form of a sheath of palms in bronze tied with white silk ribbons, which bear the name of the Association and a red cross. On an application from Viscount Burnham, Chairman of the Edith Cavell Memorial Committee, praying the Westminster City Council to take over the maintenance of the statue, the Committee report that the City Council has no authority to incur expenditure for this purpose, but that it is in the power of His Majesty's Commissioner of Works to take it over. As the memorial is a national one, the Committee are of opinion that the Government should undertake its care and preservation.

The *Daily Telegraph* Shilling Fund for Nurses has gone on steadily mounting up its total during the past week, and now stands at 237,631 shillings. Miss Beeman has collected 700 shillings, the *Leicester Mail* has sent in another generous contribution amounting to over 2,000 shillings, and the contributions from officers and sergeants run the efforts of the nurses themselves hard and testify to the continued interest taken in the Fund in military circles. It is pleasant to realise that many members of the public, whose connection with nurses has up to the present time been of a purely formal nature, have learned through this Fund some of the aims

**ROUND THE HOSPITALS—(*continued*).**

and ideals which actuate the nursing profession. In this respect it is likely to have a lasting effect for good.

---

St. Dunstan's Hostel for Blinded Soldiers is making known its needs and its triumphs in a novel war. On April 17 a party of ladies started out in a caravan, under the auspices of the National Institute for the Blind, on a hawking tour round Hertfordshire. The van is furnished with a variety of useful and ornamental things made by the blind students at St. Dunstan's, and wherever it halts talks will be given to country audiences on the work of the National Institute and the many activities of St. Dunstan's. Among the ladies who are lending their services on this interesting excursion are Miss V. Collum, Dr. Eleanor Hodson, M.B., and Mrs. Alison, all of whom are connected with the Scottish Women's Hospital staff. The tour will end on May 1, and, if successful, will perhaps lead to a further development of the caravan movement for propaganda purposes.

---

The Salaries Amending Ordinance which is coming up for approval before the Cape Provincial Council is a measure pressed forward by the Trained Nurses' Association, and is justly claimed as " one of the biggest successes it has so far achieved." It is coupled with an ordinance which makes " special provision in relation to nurses who resigned or left their posts and enrolled for military service during the European War." The new scale of salaries shows a marked advance on the former one. There are four grades of hospitals, Grade 1 containing over 400 beds and Grade 4 twenty-five beds or under. Matrons' salaries in Grade 1 start at £312 10s. and rise to £437 10s.; in Grade 4 they start at £125 and rise to £175. Sisters start at £112 10s. and rise to £150, staff-nurses at £84 rising to £104. Probationers in their third year have £36. The allowance for board and lodging to a district nurse is increased from £60 to £90. Allowance for uniform to matrons and district nurses rises from £12 to £20.

---

The fate of the West Kent Hospital, Maidstone, may be said largely to hinge on the efforts which are to be made by women in their campaign week, April 25 to May 1. A large and representative gathering of ladies at the Town Hall on April 12 was presided over by Mrs. Cornwallis, who set the facts clearly before her hearers and was supported by Mr. Gerald Mercer. The hospital is terribly cramped in its operations for lack of space. There is a waiting-list of ninety surgical cases, for whom delay is a matter of life and death, while the available medical accommodation is grossly inadequate. There is a strong desire in some quarters to get the hospital removed to another site outside the town, but near by, where there could be gardens for convalescents, open-air treatment, and an opportunity of growing produce. A very large sum of money would be required for this, but if the Women's Week testifies to a hearty desire on the part of the Maidstone people to make their hospital worthy of the town, the achievement will be brought within measurable distance. The appeal is supported in the *South Eastern Gazette* by an admirable portrait of Miss Furze, who was appointed matron last year on the conclusion of a spell of war work as charge-sister of the military block at University College Hospital, where she had previously been assistant-matron. Miss Furze is one of the keenest workers in the extension, or removal, campaign and does much in a quiet way to stimulate public interest in the work.

---

Nurses at the Antipodes are taking eagerly to the title of sister, or nursing sister, instead of nurse for fully-trained nurses. At a council meeting of the Royal Victorian Trained Nurses' Association held last February in Melbourne, the question of nomenclature came up again, and it was decided that as the members at the general meeting unanimously expressed the wish that nurses who are doing private nurses' work might use the title of sister, they may be permitted to do so. The badge of the Association is considered a sufficient distinction, and the use of a special uniform unnecessary. In this country the authorities are still hanging back, fearing possibly that confusion may ensue if private and district nurses use the title consecrated by long usage to those who hold posts of authority in the services and in institutions. Seeing that the sisters of wards do not as a rule make use of their distinctive title outside the walls of the institution, the fear of difficulty arising on that score appears to us ill-founded. The urgent need for some better description of the fully-trained than R.N. will, we trust, lead before long to decided action.

---

A remarkable amendment has been made to the statutes in Canada providing that hospitals for the insane should for the future be known as " Ontario Hospitals," on the ground that a stigma attached to persons received in institutions expressly mentioning the cause of their complaint. In spite of this change of name, however, Canadian women do not take kindly to work among mental patients, and these Ontario Government Hospitals, having advertised for probationers vainly for months past, have failed to secure them because, of course, the real nature of the work to be undertaken had to be disclosed to the numerous applicants. In these circumstances the Provincial Secretary, the Hon. H. C. Nixon, was induced to try whether the same prejudice against the mentally afflicted operated in Scotland. The vacant posts were duly brought to the notice of the public in that country; a Canadian nursing sister was charged with the duty of selecting the candidates, and within a short time 100 Scottish probationers were secured for work in the Ontario Hospitals.

---

It has been computed that each year India loses two million babies, while many others survive only to grow up weakly and feeble owing to malnutrition and bad hygienic surroundings in infancy. It is the intention of the Child Welfare Association to combat

**ROUND THE HOSPITALS**—(*continued*).

these evils by spreading knowledge and by training up skilled workers. The Association has founded a school in Delhi for the training of health and maternity supervisors, and it proposes to lend encouragment and financial assistance to local authorities which desire to maintain such supervisors in various cities. The exhibition held recently was divided into seven main sections, dealing with pre-maternity, maternity, infant welfare, childhood, first-aid, home-nursing, domestic hygiene and sanitation. There were also pathological and poster exhibits. The Calcutta Corporation sent an exhibit showing maternity work carried on under its auspices. A prize was offered by Lady Willingdon for the best essay on "How to Organise a Maternity and Child Welfare Exhibition in my own District," to be written by a genuine visitor to the exhibition. Another prize has been offered to the schoolgirl who writes the best essay on "What I learned from the Exhibition."

---

The excessive cost of clothing in America has led to a general movement against buying costly garments, which is having amusing results. Clubs are being formed in every city to promote the self-denying ordinances, and the members, who belong to all ranks of society, have adopted a plain uniform in protest, the men a blue overall, the women a blue gingham apron. The motto is: "Overalls over all," and the movement is spreading like wild-fire. There can be little doubt it will prove effective in bringing down prices, and it may conduce to simplicity in garb, though we doubt whether the promoters of "Old Clothes and Patched Clothes Associations," who are also busy, will have much success now that war is over.

---

A painful impression has been created in nursing circles in Paris by the discovery in a wood near Versailles of the dead body of Miss Ellen Appel, a member of the American Red Cross. The deceased nurse disappeared a few days ago on the eve of her departure for Vienna, where she had undertaken work under the Society of Friends. Sealed letters addressed to friends and members of her family were found on her body, and as there were no external marks of violence it is conjectured that she may have taken poison. In a letter written to a friend Miss Appel spoke of mental depression, and it is feared that her recent labours in the Marne district may have undermined her strength. A *post-mortem* examination will be made.

---

The Committee of the Royal Southern Hospital, Liverpool, has done well in securing £7,700 towards the sum of £12,000 required to provide additional accommodation for the nursing staff. The appeal was only issued at the beginning of March, and we hope that the next few weeks will see the remaining £4,300 safely banked to the hospital's credit. The additions will enable the nursing staff to be increased and their working hours and conditions to be improved in accordance with the better standard now generally recognised as desirable in the interests not only of the workers, but of the patients and the public.

---

The new schoolrooms at the Heritage Craft Schools for Cripples at Chailey, opened on April 17, are a tribute to the success of the new doctrines of Madame Montessori. Signorina Anna Maccheroni attended on the occasion in the absence of Madame Montessori, and was received by Dr. O. W. Kimmins, Chief Inspector to the London County Council. In declaring the schools open she observed that every Montessori school which is opened becomes a centre for Montessori propaganda. The Heritage Craft Schools, where suffering children are taught and tended by highly-skilled nurses, are peculiarly fitted to demonstrate the value of the Montessori method, which aims at externalising the minor forces of life and giving them expression.

---

The value of the Red Cross Organisation in time of peace is well illustrated by the reports issued by the American Red Cross of their home activities during 1919. The extent of the area over which the organisation operates is well illustrated by the disasters in which its aid was invoked. These included thirty-seven tornadoes, two severe storms, two earthquakes, six floods, ten fires and explosions, three mine disasters, two racial riots, and a serious drought. Collectively these disasters resulted in 650 deaths, nearly 2,000 persons injured, and 50,000 rendered homeless. In every case the trained Red Cross workers proved their efficiency. Starvation and hopeless wretchedness would have overtaken the victims of these disasters had not emergency relief been at once available through Red Cross sources. In some instances, owing to the application of inspired voluntary help, good was actually brought out of evil and a new community spirit was born among groups of persons divided by race and creed. Red Cross work over here in peace-time is unlikely to show these sensational developments, but it is equally necessary to combat the evils resulting from age-long prejudices and abuses.

---

Colonel Pringle has been advising the Railway Company to have an outfit of first-aid requisites, bandages, etc., supplied for use in the brake-van of all passenger trains, with the emergency tools and fire appliances usually carried. On the occasion of a collision, which took place on the Great Central Railway this winter between Mottram and Godley East, it transpired that no first-aid materials were at hand, and the absence of any bandages to dress the wounds of the injured caused much unnecessary suffering. Ambulance stores are generally to be had at stations, and are always sent through on the break-down trains, but the need is for something which can be put to immediate use by doctors or nurses on the spot.

